# Evaluation of pre-heated composite resins with soft-start polymerization and conventional composite restorations in class-I carious lesions – A randomized clinical trial

**DOI:** 10.1016/j.heliyon.2024.e30794

**Published:** 2024-05-08

**Authors:** Niral Kotecha, Nimisha C. Shah, Namita N. Gandhi, Priya Porwal, Ajinkya M. Pawar, Novaldy Wahjudianto, Dian Agustin Wahjuningrum, Suraj Arora, Mohmed Isaqali Karobari

**Affiliations:** a Department of Conservative and Endodontics, K M Shah Dental College and Hospital, Sumandeep Vidyapeeth, Vadodara, Gujarat, India; b Department of Conservative and Endodontics, Nair Hospital Dental College, Mumbai, Maharashtra, India; c Department of Conservative Dentistry, Faculty of Dental Medicine. Universitas Airlangga, Surabaya, Indonesia; d Department of Restorative Dental Sciences, College of Dentistry, King Khalid University, Abha, Saudi Arabia; e Dental Research Unit, Centre for Global Health Research, Saveetha Medical College and Hospital, Saveetha Institute of Medical and Technical Sciences, Chennai, Tamil Nadu 600077, India

**Keywords:** Composite resins, Direct composite, Heating, Light curing of dental resins, Occlusal caries

## Abstract

**Background:**

By increasing fluidity and conversion, pre-heated composites enhance adaptability and strength, while soft-start polymerization decreases internal stresses.

**Aim:**

Over a period of a year, this split-mouth design, randomized controlled clinical trial (RCT) compared pre-heated composites with soft-start polymerization to conventional composites in class-I lesions, with the goal of improving restoration outcomes.

**Methods:**

and Findings: Immediately following ethical approval and registration with CTRI, 37 patients with in-formed permission who met specified inclusion and exclusion criteria for class-I lesions were chosen for enrollment. Using a 1:1 ratio, teeth were randomly assigned to Group-A (pre-heated composite with soft-start polymerization) or Group-B (traditional composite restoration). At three-time intervals, the evaluation was blinded and calibrated using Modified United States Public Health Service (USPHS) criteria: baseline, six-month, and one-year marks. Statistical analysis was performed using SPSS 21.0 and the Mann-Whitney *U* test for inter-group comparisons and the Friedman test for intra-group comparisons.

**Interpretation:**

Pre-heated composites with soft-start polymerization performed better in terms of marginal adaptation with a statistically significant difference (p = 0.019) and in terms of color match they performed better clinically (p = 0.062) at 12 months. Other variables like marginal discolouration, sec-ondary caries, anatomic form, post-operative sensitivity, surface texture and retention showed no statistically significant difference (p < 0.05). Pre-heated composites with soft-start mode performed marginally better than nanofilled composites. However, both techniques can be used to successfully restore simple class-I carious lesions.

## 1 Introduction

Composite resins stand as the predominant materials employed in the realm of direct aesthetic restorations, serving as the material of choice in this domain. Within the composition of composite resins, the individual constituents collectively contribute to conferring a remarkable combination of rigidity and durability, thereby endowing the restorations with robustness. Concurrently, these constituent elements preserve an optimal degree of fluidity, ensuring effortless and uncomplicated application during the restorative process [1]. Recent advances in the science of composite materials, together with the refining of clinical treatment techniques that address their shortcomings, have resulted in the massive popularity of direct composite restorations [2]. Polymerization shrinkage and marginal adaptation are still inherent drawbacks of direct composites among many other drawbacks [3]. In response to these obstacles, manufacturers have dedicated the past decade to enhancing the mechanical attributes of composites, thereby fostering a greater inclination among practitioners to utilize composites more frequently in the restoration of posterior dental structures. These advancements have been strategically targeted at refining the microstructural aspects of the material. These encompass a meticulous consideration of the monomer composition, dimensions, morphology, and dispersion patterns of the inorganic filler particles, with a particular emphasis on optimizing the proportion of filler loading [4]. The majority of issues have been effectively addressed in contemporary composite materials such as nanofilled composites [5]; nonetheless, challenges remain, particularly pertaining to polymerization shrinkage and the precision of marginal adaptation. These problems are addressed by using flowable composites under packable composites or preheating the composites to reduce their viscosity [6]. Flowable composites, because of their high fluidity, promote adaptation and function as stress absorbers; nevertheless, higher polymerization-induced stress is expected compared to conventional resin due to lower filler concentration [6,7]. Research has demonstrated that preheating of resin-based composites increased their marginal adaptation as a result of increased fluidity allowing it to flow precisely onto the walls of the cavity, internal angles, and narrow or undercut sections of the defects making it a beneficial technique [2]. Moreover, resin composite preheating enhances the maximum rate of polymerization and monomer conversion which in turn enhances their mechanical and physical properties [8].

Curing lights and light polymerization techniques have also remarkably improved, aimed at increasing the depth of cure and reduction of polymerization shrinkage stresses [9]. The utilization of Light Emitting Diode (LED) technology provides advantages that encompass a prolonged operational lifespan characterized by consistent light intensity, limited heat generation, and commendable resilience against shock and vibration [10]. These days, third generation LED curing lights are employed which are known to effectively cure a variety of composites with additional photo initiators, such as 2, 4, 6- trimethyl benzoyl-diphenyl phosphine oxide (TPO). They combine LED chips with outputs that are close to the range of camphorquinones and the 400–410 nm range, which is close to TPO's absorption range, enabling its more effective activation [11]. Despite these notable progressions, the occurrence of polymerization shrinkage in composites persists, attributed to the emergence of post-gel polymerization strains at the interface between the composite and the tooth. This phenomenon arises from the cessation of flow, which becomes inadequate in mitigating the stresses induced by shrinkage [12]. Soft start polymerization, which involves using a lower intensity of curing light initially, followed by a higher intensity, has demonstrated a reduction of internal stresses in composites and improved marginal adaptability by extending the pre-gel phase, which alleviates stresses built within the structure [13].

There are several in-vitro and clinical studies demonstrating the benefits of pre-heated composites and the use of soft-start polymerization [[14], [15], [16]]; however, there are no clinical studies illustrating the benefits of combining the two approaches. Hence, this study was designed to compare and evaluate the one-year clinical performance of pre-heated composite resins with soft-start curing mode and conventional composite restorations in simple Class I lesions, with the null hypothesis that there would be no distinctions in the clinical performance of conventional composite restorations and pre-heated composite restorations with soft-start polymerization in simple Class I lesions at baseline (immediately after finishing and polishing of the restorations), 6 months and 1-year intervals.

## 2 Material and methods

### 2.1 Ethical approval statement

**Fig. 1:**
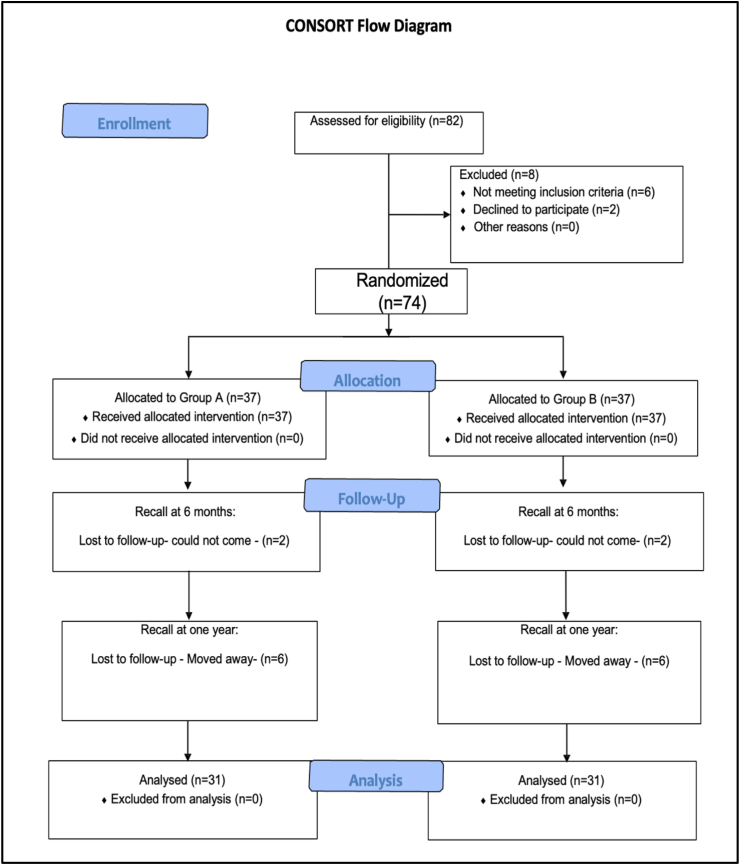
CONSORT flow diagram of the clinical trial.

The Institutional Ethics Committee provided prior ethical approval (SVIEC/ON/DENT/BNPG20/D21035) and the protocols were entered into the www.ctri.nic.in database (CTRI/2021/06/034,364). The reporting of this study was done according to Consolidated Standards of Reporting Trials (CONSORT) guidelines (Fig. 1).

### 2.2 Patient selection

#### 2.2.1 Inclusion criteria

Patients between 18 and 60 years of age with at least two maxillary or mandibular molars with moderate stage caries-up to ICDAS score 4 and RB Radiographic staging (i.e.- radiolucency reaching the middle 1/3 of dentin) were included for the study. Intraoral Periapical radiographs were taken by paralleling technique using a long cone for the confirmation of the extent of the lesion. Radiographs showing radiolucency involving enamel and dentin (up to RB score) and not involving pulp or the interproximal areas or buccal and lingual surfaces were selected. Also, those teeth were subjected to thermal and electric pulp testing and only teeth giving signs of reversible pulpitis were included. Additionally, teeth indicating secondary decay or fractured fillings that gave signs of reversible pulpitis and after radiographic confirmation of the extent of defect, were included in the study.

#### 2.2.2 Exclusion criteria

Patients with severe or chronic periodontitis, heavy bruxism, malocclusion, rotated, teeth with attrition or with pulpal and/or periapical pathology, developmental anomaly or pathology, and teeth which had to be used as an abutment were excluded from the study. Patients with Oral Hygiene Index- Simplified (OHI–S) score ranging from 3.1 to 6.9 were excluded from the study.

### 2.3 Sample size estimation

According to research done by Ayub et al. [17], a minimal sample size of 74 teeth (37 teeth per group) was required for this study at 5 % alpha error, 95 % confidence, and 80 % power, including a drop-out rate of 20 %. The sample size calculation was done using G-Power software.

### 2.4 Randomization and allocation

The teeth were then divided by computer randomization method (www.randomizer.org) into-Group A: Composite Restoration using preheated composite resins with soft-start mode (n = 37); Group B: Conventional Direct Composite Restoration (n = 37) (Table 1). Allocation concealment was achieved using the Sequentially Numbered Opaque Sealed Envelope (SNOSE) approach with a 1:1 allocation ratio, and the processes of sequence creation and allocation concealment were accomplished by a researcher who was not engaged in any of the experimental stages. Since this was a double-blind experiment, neither the patient nor the assessors knew which therapy group was allocated to which tooth.

**Table 1:** Details of the materials used for the study.

Product name	Manufacturer	Components	Batch	Expiry	Shade
3 M ESPE Filtek Z350 Xt	3 M, USA	Bis-GMA, UDMA, TEGDMA, Titanium oxide, Barium fluoride, Aluminium silicate glass.	E7501	02/2023	Translucent, enamel A2,A1, Body A2, A1
**Local anaesthetic agent**	Zinocaine-A (Laborate Pharmaceutical India Ltd)	Lignocaine hydrochloride, Adrenalin bitartrate	JZA1-006	08/2022	–
**Etchant**	D-tech Dental technologies, India	37 % Phosphoric acid	EG010720	07/2022	–
**3M™ Single Bond Universal Adhesive**	3 M, USA	MDP Phosphate Monomer Dimethacrylate resins HEMA Vitrebond Copolymer Filler Ethanol Water Initiators Silane	1912231	12/2022	–
**Calcimol LC**	NDT-VOCO	Calcium hydroxide, methacrylates, butylated hydroxyl toluidine and amines.	1824207	06/2023	–
**Caries indicator dye**	Prime Dental	Propoane-1,2-diol Dye	18040301	04/2023	–
Kolor + Plus Resin color modifier	Kerr Dental				

### 2.5 Clinical procedure

The entire restorative procedure for both the groups was performed by the primary investigator (single operator) which was commenced with dental prophylaxis and shade selection by composite button technique where small composite buttons of dentin and enamel shades were placed on the occlusal tooth surface and a black and white image was captured to eliminate hue and chroma with a Canon Digital Single-Lens Reflex (DSLR) camera (Oita, Japan) with a 100 mm macro lens and ring flash followed by single tooth rubber dam (Nic tone, MDC Dental, Mexico) isolation. Caries excavation was done using a carbide bur (Mani, Japan) and dental spoon excavators (Hu-friedy, Rockwell, Chicago). Presence of any remaining carious lesion was confirmed by the application of caries indicator dye (Kuraray Dental INC., Tokyo, Japan). Pulp protection was performed using light-cured calcium hydroxide (Calcimol LC, Voco, Germany) (if necessary), which was followed by selective enamel etching was done with 37 % phosphoric acid (D-Tech, India) for 20s and rinsed for 1 min. The universal bonding agent (Single bond universal adhesive, 3 M ESPE, USA) was then applied on to the cavity walls with an applicator tip and light cured for 10s using 3rd generation LED curing light (Orikam HealthCare, China). The light intensity of the curing light was checked before starting each patient using a radiometer. The teeth were then classified as Group A or Group B.

### 2.6 Group A: composite restoration using preheated composite resins with soft-start mode (n = 37)

**Fig. 2:**
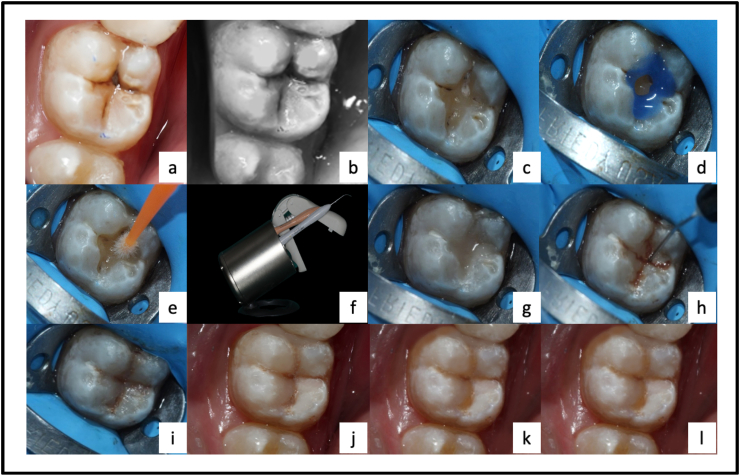
Composite Restoration using preheated composite resins with soft-start mode: (a) Pre-operative clinical photograph, (b) Shade selection by composite button technique, (c) Caries excavation under rubber dam, (d) Selective etching, (e) Application of bonding agent, (f) Preheating of composite compules and instruments in composite warmer at 60 °C, (g) Body shade application, (h) Tint application, (i) Enamel shade application, (j) Post-operative photograph, (k) 6 months follow-up, and (l) 1-year follow-up photograph. (For interpretation of the references to color in this figure legend, the reader is referred to the Web version of this article.)

Compules of the nanofilled composite resin (3 M ESPE Filtek Z350 Xt, 3 M, USA) in a dispenser gun were heated in the composite warmer (Waldent Innovations Private Limited, India) for 10 min at 60 °C. Each increment (body shade followed by enamel shade) of the heated composite resin was applied immediately into the isolated cavities in successive cusp build-up technique and cured using 3rd generation LED curing light (Orikam HealthCare, China) in soft start/ramp mode (600 mW/cm2 for 5s followed by 1000 mW/cm2 for 15s) such that the tip of the light was held as closely as possible to the teeth without actually touching them. Before application of enamel layer, brown tint (Kolor + Resin color modifier, Kerr Dental, USA) was applied and light cured (Fig. 2a-l).

### 2.7 Group B: composite restoration using conventional direct composite (n = 37)

Each increment (body shade followed by dentin shade followed by enamel shade) of nanofilled composite (3 M ESPE Filtek Z350 Xt, 3 M, USA) at room temperature (28 °C) was placed into the isolated cavities in cusp-by-cusp incremental manner and cured using 3rd generation LED curing light (Orikam HealthCare, China) in normal mode at 1000 mW/cm2 intensity such that the tip of the light was held as closely as possible to the teeth without actually touching them. Before application of enamel layer, brown tint (Kolor + Resin color modifier, Kerr Dental, USA) was applied and light cured.

Finishing and polishing of all the teeth was carried out by using green silicon carbide stones for adjusting, white aluminum oxide stones (Composite finishing kit, Shofu Dental INC., Japan) for fine finishing and polishing along with fine abrasive paste (Directdia diamond polishing paste, Shofu Dental INC., Japan). The occlusion with the opposite arch was checked articulating papers (Arti-check micro thin, Bausch Articulating Papers, Inc., USA) of 40μ.

### 2.8 Evaluation

Two evaluators, who were blinded, assessed and evaluated for Marginal Discoloration, Marginal Adaptation, Secondary Caries, Surface Texture, Anatomic form, Post-operative sensitivity, color match and Retention according to Modified United States Public Health Service (USPHS) criteria (Table 2) [18] at the baseline (immediately after finishing and polishing of the restorations), at 6 months and 1-year periods. Weighted Kappa (KW) with linear weights was used to test inter-evaluator reliability with a value of 0.85.

**Table 2:** Modified USPHS scoring criteria (Bayne SC, Schmalz G) [18].

Marginal Discoloration	Alpha: No marginal discoloration Charlie: Marginal discoloration present
Marginal Adaptation	Alpha: Margin not discernible, probe does not catch Bravo: Probe catches on margin but no gap, dentin or liner exposed Charlie: Probe catches on margin and gap on probing, dentin or liner exposed Delta: Restoration fractured or missing
Secondary Caries	Alpha: No evidence of caries Charlie: Caries is evident contagious with the margins
Surface Texture	Alpha: Smooth, glazed, or glossy surface Bravo: Slightly rough or dull surface Charlie: Surface with deep pores, cannot be refined
Anatomic form	Alpha: correct contour Bravo: slightly under- or over contoured Charlie: distinctly under- or over contoured Delta: restoration fractured or mobile
Postoperative sensitivity	Alpha: No postoperative sensitivity Bravo: Postoperative sensitivity Charlie: Postoperative sensitivity with Treatment need
Color match	Alpha: Matches tooth Bravo: Acceptable Mismatch Charlie: Unacceptable Mismatch
Retention	Alpha: No loss of restorative material Charlie: Loss of restorative material (Partial/full)

### 2.9 Statistical analysis

The statistical analysis was carried out with the Mann- Whitney *U* test for the inter-group analysis and Friedman test for the intra-group analysis using Statistical Package for Social Sciences (SPSS) version 21 (IBM Corp., Armonk, NY, USA) with P < 0.05 considered as significant and a 95 % confidence interval.

## 3 Results

Of the 32 patients treated, 17 were males, while 15 were females. The age of 4 patients was between 46 and 60 years, age of 13 patients was between 31 and 45 years, while age of rest 15 patients was between 18 and 30 years. Of the 74 class I lesions 28 were in maxilla and 46 were in mandible. The dropout percentage was 16.21 % for groups A and B which was compensated under 20 % of dropouts included in the sample size and thus did not affect the power of the study. Fig. 1 shows the CONSORT flow diagram for this study.

On intra-group analysis using Friedman test (Table 3), both the groups showed some amount of deterioration in terms of all the parameters from baseline through the periods of 6 months and 12 months. For Group A, there was a statistically significant difference in terms of marginal discoloration (p = 0.001), surface texture (p = 0.005), and color match (p = 0.018), by the end of one-year evaluation. The p values for secondary caries and post-operative sensitivity were 0.10, 0.135 respectively with no statistically significant difference. There was no change observed in terms of marginal adaptation, anatomic form and retention at the end of one year. For Group B, there was a statistically significant difference in terms of marginal discoloration (p < 0.001), marginal adaptation (p = 0.005), surface texture (p = 0.002), color match (p = 0.001), at the end of one-year evaluation. The p values for secondary caries, anatomic form, post-operative sensitivity and retention were 0.223, 0.368, 0.097, 0.05 and 0.368 respectively which were statistically not significant.

**Table 3:** Intra-group analysis of the groups using Friedman's test at baseline, 6 months, 1-year intervals.

		Baseline			6 months			12 months			p-value
		Alpha	Bravo	Charlie	Alpha	Bravo	Charlie	Alpha	Bravo	Charlie	
Group A	Marginal discoloration	37	0	0	31	0	4	22	0	9	**0.001**
	Marginal adaptation	37	0	0	35	0	0	31	0	0	–
	Secondary Caries	37	0	0	35	0	0	28	0	3	0.10
	Surface Texture	37	0	0	32	3	0	24	7	0	**0.005**
	Anatomic form	37	0	0	35	0	0	31	0	0	–
	Post-operative sensitivity	37	0	0	35	0	0	29	2	0	0.135
	Color match	37	0	0	35	0	0	27	4	0	**0.018**
	Retention	37	0	0	35	0	0	31	0	0	–
Group B	Marginal discoloration	37	0	0	30	0	5	16	0	15	**<0.001**
	Marginal adaptation	37	0	0	32	3	0	24	6	1	**0.005**
	Secondary Caries	37	0	0	34	0	1	29	0	2	0.223
	Surface Texture	37	0	0	30	5	0	23	8	0	**0.002**
	Anatomic form	37	0	0	34	1	0	30	1	0	0.368
	Post-operative sensitivity	37	0	0	34	1	0	28	3	0	0.097
	Color match	37	0	0	32	3	0	22	4	5	**0.001**
	Retention	37	0	0	35	0	0	30	0	1	0.368

On inter-group comparison using Mann- Whitney *U* test (Table 4), Group A performed better in terms of marginal adaptation with a statistically significant difference (p = 0.019) also in terms of color match, it performed better clinically (p = 0.062) at 12 months. There was no statistically significant difference in the clinical performance of Composite Restoration using preheated composite resins with soft-start mode (Group A) and Conventional Direct Composite Restoration group (Group B) in terms of marginal discoloration, secondary caries, surface texture, anatomic form, postoperative sensitivity and retention in both the techniques at the end of baseline, 6 months and 1 year. Comparison of the Time taken - Baseline (in minutes) between the two groups using Independent *t*-test, shows that time taken (in minutes) is higher in Group B group with a t value of −4.956 and is statistically significant with a p value of <0.001 (Table 5).

**Table 4:** Inter-group comparison between the two groups using Mann-Whitney *U* test at baseline, 6 months and 1-year intervals.

Evaluation criteria	Group A (Preheated composite resins and soft-start mode) N (%)			Group B (Conventional composite restoration) N (%)			*P value*	
	Baseline	6 months	1 year	Baseline	6 months	1 year	6 months	1 year
Marginal Adaptation							0.077	0.019
Alpha	37 (100)	35 (100)	31 (100)	37 (100)	32 (91.4)	24 (77.4)		
Bravo	0 (0)	0 (0)	0 (0)	0 (0)	3 (8.6)	6 (19.4)		
Charlie	0 (0)	0 (0)	0 (0)	0 (0)	0 (0)	1 (3.2)		
Color match							0.077	0.064
Alpha	37 (100)	35 (100)	27 (87.1)	37 (100)	32 (91.4)	22 (71)		
Bravo	0 (0)	0 (0)	4 (12.9)	0 (0)	3 (8.6)	4 (12.9)		
Charlie	0 (0)	0 (0)	0 (0)	0 (0)	0 (0)	5 (16.1)

**Table 5:** Comparison of time taken to perform restoration of preheated composite resins and soft-start mode group (Group A) and conventional composite restoration group (Group B) using Independent-t test.

	Group A (n = 37)	Group B (n = 37)	t	P VALUE
	Mean ± sd	Mean ± sd		
Time taken – Baseline (in minutes)	48.24 ± 8.52	57.03 ± 6.61	−4.95	**<0.001**

## 4 Discussion

Occlusal caries affecting permanent molars represent a commonly encountered issue, often necessitating direct restoration. In such cases, nanofilled composites, acknowledged as the benchmark for posterior restorations, have conventionally been selected as the preferred restorative material. This rationale underpins their selection as the material of choice for the present study. The incremental layering of composites not effectively diminishes the Configuration-factor, thereby mitigating the impact of polymerization shrinkage-induced stresses but also contributes to the meticulous replication of the natural tooth's optical characteristics [19]. Thus, the incremental layering technique was used for all the restorations in this study. The disadvantages of self-etch adhesives include bond strengths to enamel that are typically lower, poor bond strengths to self-cure/dual-cure composites and cement, and long-term bond to dentin being susceptible to hydrolysis. The method which incorporates the best of both these techniques is the selective etch technique. Advantages of Selective-Etch include high bond strengths to cut and uncut enamel, less technique sensitive, and less risk of post-op sensitivity. Hence, selective etching technique was used in our study [20,21].

Numerous investigations, both in-vitro and in-vivo studies, have consistently yielded findings indicating that the application of heat to composites yields favourable outcomes. This practice has been associated with enhanced marginal adaptation, optimal wetting characteristics facilitated by heightened fluidity, augmented levels of monomer conversion leading to superior microhardness, enhanced flexural strength, increased rigidity, elevated resistance against deterioration, and ultimately, an improved overall clinical performance [[22], [23], [24]]. The underlying reasoning for these alterations is rooted in the observation that, on a microscopic scale, the particles comprising the composite material gain an elevated level of kinetic energy, enabling them to exhibit swifter and more energetic movement compared to their counterparts operating under standard temperature conditions [25]. The extent to which the conversion of monomeric carbon-carbon double bonds into polymeric carbon-carbon single bonds takes place experiences a substantial enhancement as a result of the heightened mobility exhibited by monomers and free radicals within the composite material when subjected to elevated temperatures, reaching up to 70 % (with an average of 68.3 % at 60 °C), compared to the baseline room temperature value of 52 %. This augmentation in conversion efficiency contributes to the notable improvements in material properties, primarily attributed to the intensified cross-linkage mechanisms [26]. Hence, this technique of preheating composites was utilized as a part of one comparison group.

Within the realm of existing literature, the customary temperature range for preheating is commonly documented to fall between 54 and 68 °C [27]. This particular range is widely acknowledged as safe, as it poses no detriment to the health of the underlying pulp tissue. Notably, the temperature of the composite material, following its preheating process and subsequent insertion into the prepared cavity, undergoes a rapid decline. This temperature reduction amounts to a substantial 50 % decrease within a mere span of 2 min subsequent to removal from the heating unit [28]. Furthermore, it is important to note that the residual dentin assumes the role of a proficient thermal insulator, effectively limiting the extent of temperature elevation within the pulp. Consequently, even when the composite resin is subjected to a temperature of 60 °C, the resultant increase in pulpal temperature remains significantly modest, not exceeding 0.8 °C [29]. This observed temperature rise stands well below the critical threshold of 5.5 °C, which is recognized as the potential point of concern for potential harm to the delicate pulp tissue in human teeth [30]. Hence, composites were pre-heated at 60 °C considering it to be a safe and beneficial technique.

Nonetheless, as the degree of conversion experiences an upward trajectory, an accompanying escalation in polymerization shrinkage stress becomes evident. This phenomenon can be attributed to the sudden accumulation of stress within the composite material, an outcome of the heightened polymerization rate coupled with an earlier attainment of the gel point. This increase in shrinkage stress is compounded by the presence of pronounced thermal shrinkage, which occurs when pre-heated composites cool down to the prevailing ambient temperature [15].

In an effort to mitigate the stresses arising from the shrinkage of resin composites, various curing strategies have been suggested. Among these techniques, the implementation of soft-start polymerization stands out, wherein the initial phase of polymerization, known as the pre-gel stage, is intentionally extended. This deliberate extension serves as a mechanism to alleviate and dissipate the resulting stresses, thereby contributing to a reduction in overall shrinkage stress within the composite material [13]. Hence, a combination of preheating and soft-start polymerization was employed to achieve a higher degree of conversion and reduced post-gel shrinkage strain.

The results of this study demonstrated that Group A performed better in terms of marginal adaptation with a statistically significant difference (p = 0.019) also, in terms of color match it performed clinically better (p = 0.062) at 12 months, although not statistically significant. This advantageous outcome observed within Group A's marginal adaptation can likely be attributed to the effect of composite preheating, which confers heightened fluidity to the material. Consequently, the heated composite demonstrates a heightened ability to precisely adapt to the intricate contours of the cavity walls, even accommodating undercuts with minimal interstice, thereby facilitating a more optimal marginal adaptation. Furthermore, the utilization of the soft-start polymerization mode, as adopted in Group A, contributes to a reduction in internal stresses within the composite. This reduction occurs by deliberately prolonging the post-gel phase, granting additional time for the composite material to fluidly flow and adeptly conform to the margins of the cavity. This extended adaptability augments the composite's capacity for achieving improved marginal adaptation. Notably, the outcomes of this study align cohesively with the conclusions drawn from prior in-vitro investigations conducted by Nívea Regina Fróes-Salgado et al. [31], and Ernst et al., [32].

Upon the culmination of the 12-month assessment period, Group A exhibited a superior level of color stability when compared to Group B. While this disparity did not yield statistical significance (p = 0.064), it remained of notable clinical relevance. Color stability pertains to a dental material's capacity to maintain its initial color over time, and this attribute is influenced by a spectrum of inherent and extrinsic elements. Among the intrinsic factors, physicochemical discoloration reactions transpire within the composite matrix, manifesting in both superficial and subsurface layers, often caused by sources such as ultraviolet (UV) radiation, thermal fluctuations, and humidity. In contrast, extrinsic factors come into play due to the accumulation of dental plaque and staining agents, and are also contingent on the intensity and duration of the polymerization process. Environmental factors, inclusive of ambient conditions, UV irradiation, temperature, moisture, and the presence of pigmented food substances, further contribute to the color stability. This divergence in color stability can be ascribed to the increased degree of conversion and the concurrent enhancement of the physical characteristics of the resin composite within Group A. These improvements collectively result in a reduction in the absorption and infiltration of colorant solutions, thereby contributing to enhanced color stability over time. This outcome aligns harmoniously with the conclusions drawn from investigations undertaken by Darabi et al. [15], and Sousa et al. [33], both of which concurred that the preheating approach effectively enhances the color stability of composite resin materials.

In addition to the aforementioned advantages, pre-heated composites have been found to exhibit exceptional sculpting properties owing to their reduced viscosity, thereby resulting in reduced working time. Consequently, Group B, which employed conventional non-preheated composites, necessitated a lengthier procedural timeline as compared to Group A. This discrepancy in working time achieved a statistical significance (p < 0.001).

However, it is important to acknowledge certain drawbacks inherent to preheating of composites method. Notably, the temperature of the pre-heated composite experiences rapid decline once removed from the heating apparatus [28]. To counteract this, instruments were also heated to maintain the desired temperature during the application process. There was no statistically significant difference in the clinical performance of the groups in terms of other parameters of modified USPHS criteria, at the end of 1 year, this outcome led to the partial rejection of the null hypothesis.

Henceforth, it can be ascertained that Class-I cavities are amenable to successful restoration through the utilization of both preheated composite resins employing the soft-start polymerization technique, with the latter method slightly showcasing an advantageous position over the conventional approach. However, it is prudent to acknowledge certain limitations intrinsic to this study. Chief among these limitations are the relatively modest sample size and the abbreviated duration of the follow-up period. Amplifying the sample size and extending the follow-up duration would have potentially enabled a more meticulous evaluation of the restorations' enduring strength and clinical efficacy. This study encompassed participants aged between 18 and 60 years, constituting a broad demographic spectrum whose span may influence the durability of the restoration; thus, additional research is warranted involving individuals within a comparable age bracket. Furthermore, it is pertinent to recognize that this study specifically focused on simple class I lesions, utilizing nanofilled composites. To gain a more comprehensive understanding of the impact of preheating and soft-start polymerization in diverse clinical scenarios, future research endeavors should encompass an exploration of varied forms of carious lesions and diverse composite types.

## 5 Conclusion

Within the constraints of the study, simple class-I lesions can be successfully restored using both pre-heated composites cured by soft-start polymerization and conventional composites. However, pre-heated composites with soft-start polymerization performed marginally better than conventional composites with better marginal adaptation and color stability which in turn accounts for increased longevity of the composite restorations.

## Ethics statement

The Institutional Ethics Committee provided prior ethical approval (SVIEC/ON/DENT/BNPG20/D21035) and the protocols were entered into the www.ctri.nic.in database (CTRI/2021/06/034,364). The reporting of this study was done according to Consolidated Standards of Reporting Trials (CONSORT) guidelines. The informed consent was obtained from all the participants.

## Data availability

The data set used in the current study will be made available at the reasonable request.

## CRediT authorship contribution statement

**Niral Kotecha:** Writing – original draft, Investigation, Data curation. **Nimisha C. Shah:** Writing – original draft, Investigation, Data curation. **Namita N. Gandhi:** Writing – original draft, Investigation, Data curation. **Priya Porwal:** Investigation, Formal analysis, Data curation. **Ajinkya M. Pawar:** Supervision, Resources, Project administration, Methodology, Conceptualization. **Novaldy Wahjudianto:** Software, Resources, Formal analysis. **Dian Agustin Wahjuningrum:** Writing – review & editing, Supervision, Project administration, Methodology, Conceptualization. **Suraj Arora:** Visualization, Validation, Resources, Funding acquisition. **Mohmed Isaqali Karobari:** Writing – review & editing, Validation, Methodology, Conceptualization.

## Declaration of competing interest

The financial support was provided by College of Dentistry, 10.13039/501100007446King Khalid University, Abha, Saudi Arabia with Grant Number RGP.2/198/44 and Faculty of Dental Medicine. Universitas Airlangga. Indonesia that collaborate in this project. If there are other authors, they declare that they have no known competing financial interests or personal relationships that could have appeared to influence the work reported in this paper.
