# Supplementary material for: Evaluation of pre-heated composite resins with soft-start polymerization and conventional composite restorations in class-I carious lesions – A randomized clinical trial
Source: Heliyon. 2024 May 8;10(10):e30794. doi: 10.1016/j.heliyon.2024.e30794 (PMC11103470; doi:10.1016/j.heliyon.2024.e30794)
Supplement: Multimedia component 1 [file mmc1.docx]

**CONSORT Flow Diagram**

## Enrollment

## Allocation

Randomised (n=74)

Excluded (n=8)

♦  Not meeting inclusion criteria (n=6)

♦  Declined to participate (n=2)

♦  Other reasons (n=0)

Assessed for eligibility (n=82)

Allocated to Group B (n=37)

♦ Received allocated intervention (n=37)

♦ Did not receive allocated intervention (n=0)

Allocated to Group A (n=37)

♦ Received allocated intervention (n=37)

♦ Did not receive allocated intervention (n=0)

## Follow-Up

Recall at 6 months:

Lost to follow-up- could not come - (n=2)

Recall at 6 months:

Lost to follow-up- could not come- (n=2)

Recall at one year:

Lost to follow-up - Moved away- (n=6)

Recall at one year:

Lost to follow-up - Moved away - (n=6)

Analysed (n=31)
♦ Excluded from analysis (n=0)

## Analysis

Analysed (n=31)
♦ Excluded from analysis (n=0)


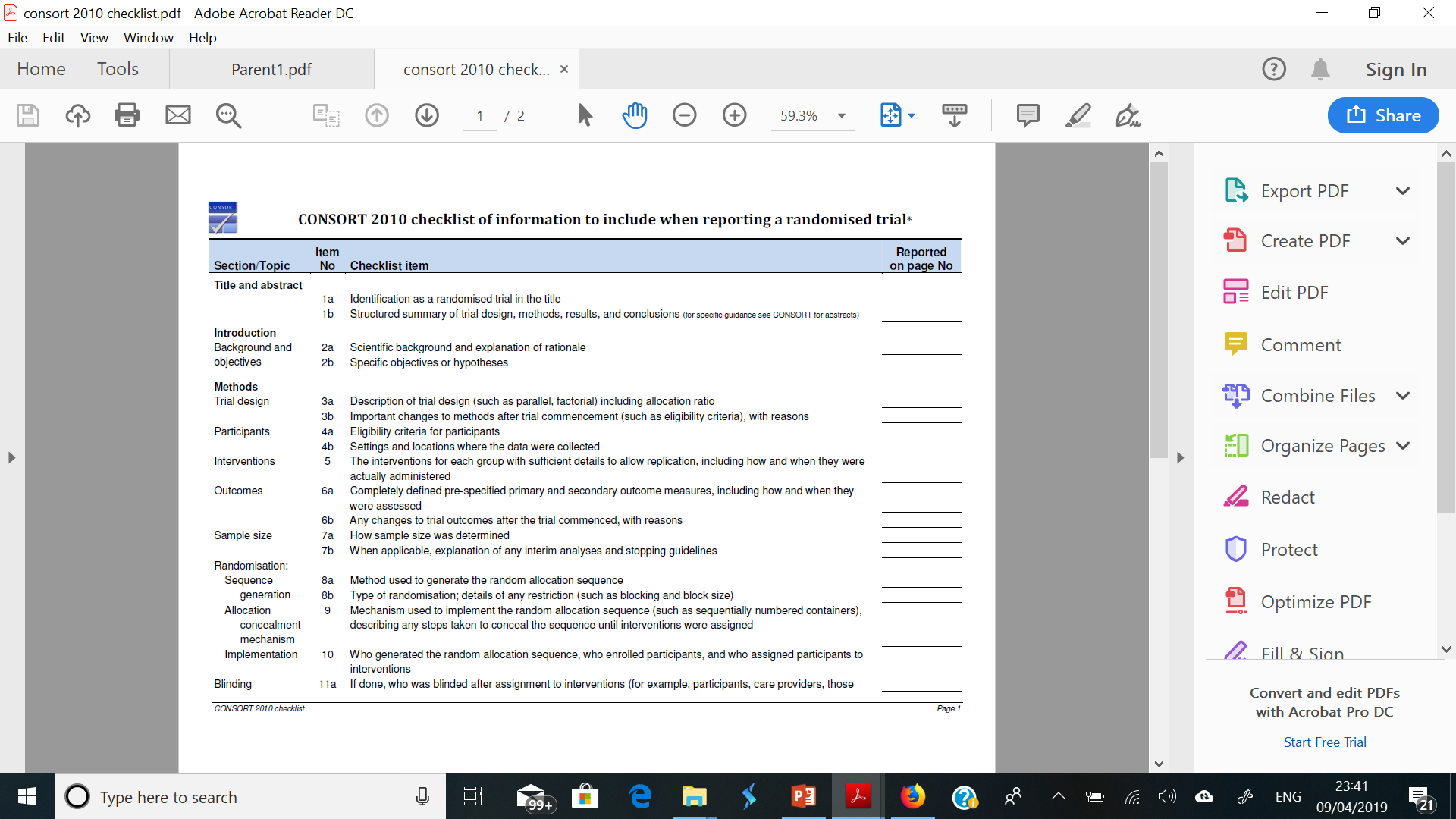


-

No

-

Yes

Yes

No

Yes

-

Yes

Yes

Yes

Yes

Yes


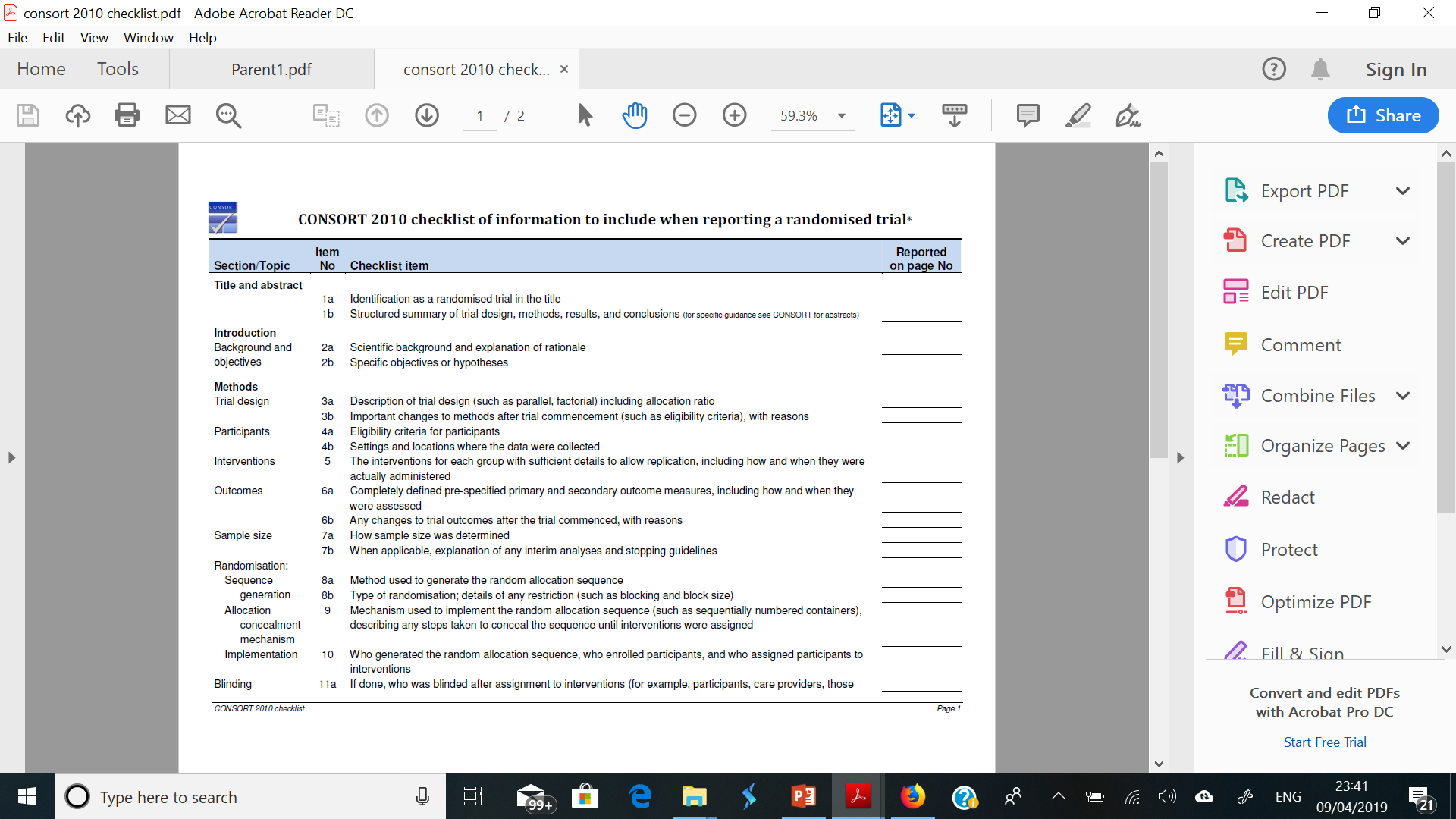


Yes

Yes

Yes

Yes

Yes


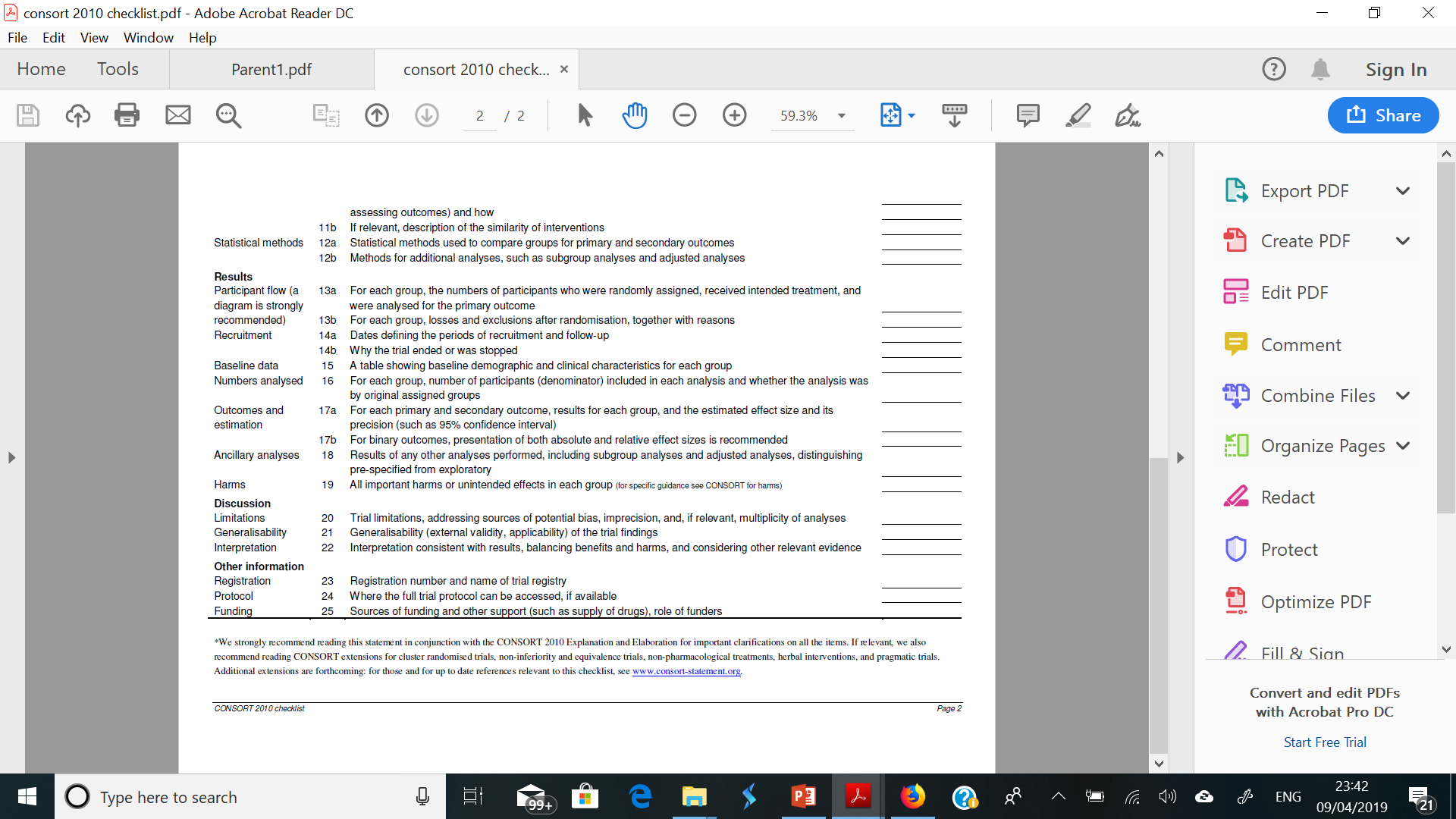


Yes

No

Yes

Yes

Yes

Yes

-

Yes


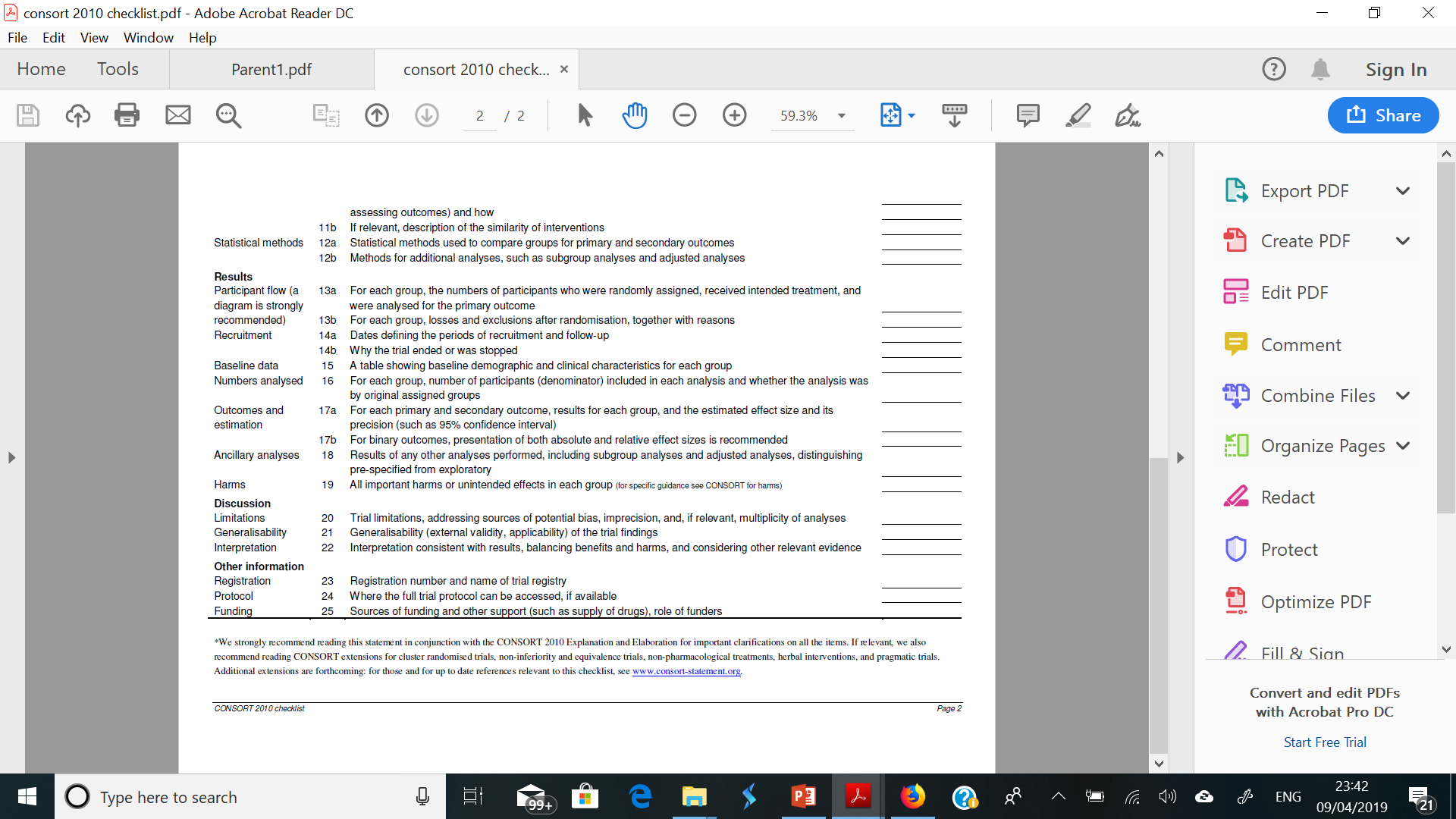


Yes

Yes

Yes

Yes

Yes

Yes
